# Therapeutic Applications of Rose Hips from Different *Rosa* Species

**DOI:** 10.3390/ijms18061137

**Published:** 2017-05-25

**Authors:** Inés Mármol, Cristina Sánchez-de-Diego, Nerea Jiménez-Moreno, Carmen Ancín-Azpilicueta, María Jesús Rodríguez-Yoldi

**Affiliations:** 1 Department of Pharmacology and Physiology, University of Zaragoza, Zaragoza 50013, Spain; ines.marmol9@gmail.com; 2 Department of Physiological Sciences II, University of Barcelona, Barcelona 08907, Spain; csanchezdg@gmail.com; 3 Department of Applied Chemistry, Public University of Navarra, Pamplona 31006, Spain; nerea.jimenez@unavarra.es

**Keywords:** Rose hip, oxidative stress, antioxidants, functional food, cancer, arthritis, diabetes, neural disorder, antimicrobial

## Abstract

*Rosa* species, rose hips, are widespread wild plants that have been traditionally used as medicinal compounds for the treatment of a wide variety of diseases. The therapeutic potential of these plants is based on its antioxidant effects caused by or associated with its phytochemical composition, which includes ascorbic acid, phenolic compounds and healthy fatty acids among others. Over the last few years, medicinal interest in rose hips has increased as a consequence of recent research that has studied its potential application as a treatment for several diseases including skin disorders, hepatotoxicity, renal disturbances, diarrhoea, inflammatory disorders, arthritis, diabetes, hyperlipidaemia, obesity and cancer. In this review, the role of different species of *Rosa* in the prevention of treatment of various disorders related to oxidative stress, is examined, focusing on new therapeutic approaches from a molecular point of view.

## 1. Methodology

The present report has adhered to systematic review guidelines. The search of each of the different parts in PubMed (http://www.ncbi.nlm.nih.gov/pubmed/) identified a total of 214 hits from 1976 to 2017.

## 2. Introduction

### 2.1. Origin of Oxidation: The Importance of Natural Antioxidants

Vegetables and fruits are an important source for therapeutic products which can prevent, relieve or cure numerous illnesses as they are an important source of phytochemicals and other bioactive compounds. Reactive Oxygen Species (ROS) are implicated in a large number of illnesses, especially chronic ones. Nitrogenous species and free radicals start chain reactions which can favour the initiation and progression of many complications in diseases [1,2]. Oxidative stress may be defined as an imbalance between ROS levels in the organism and the capacity of antioxidant mechanisms. A free radical is a species which contains one or more unpaired electrons, which makes them very reactive as they need another electron to fill the orbital and become more stable. Free radicals are formed in different ways: (i) many organic molecules (glyceraldehydes, adrenaline, l-dopa, dopamine, cysteine etc.) oxidize in the presence of O_2_ to form the superoxide radical; (ii) many of these radicals in vivo are produced by an incomplete transfer of electrons on O_2_ just before the terminal cytochrome oxidase step [3]. The superoxide radical (O_2_^•−^), which is very active, is formed when a single electron is added to the ground state O_2_ molecule. Sometimes the presence of ROS in the organism is beneficial as they are used in the immune response to kill ingested or extra-cellular bacteria. Unfortunately, ROS are not limited to this action and they can also contribute to undesired effects as they induce oxidation processes. The term “antioxidant paradox” is often used to refer that ROS are implicated in several human diseases but there is no good evidence, in human population, that large doses of dietary antioxidants have always preventive or therapeutic effects [4]. In addition, there are some environmental factors which contribute to the production of free radicals such as exposure to ultraviolet radiation, pollution and cigarette smoke. Nitrogen dioxide, one of the major oxidants in smog, is also found in cigarette smoke. Two free radicals have been found in tobacco smoke, the main radical NO^•^, found in tar, is capable of reducing oxygen to the superoxide radical [3].

The antioxidants family include a series of molecules with low oxidation potential which act by donating electrons to deactivate ROS and other free radicals that produce DNA damage and consequently can provoke tumorigenesis [3]. Antioxidants scavenge free radicals through different mechanisms like Hydrogen Atom Transfer (HAT), Single Electron Transfer followed by Proton Transfer (SET or ET-PT) and the Sequential Proton Loss Electron Transfer (SPLET) mechanism. Each one of these mechanisms presents different kinetics [5]. When the free radicals are generated in vivo, many antioxidants act in order to defend the organism from oxidative damage. In the organism there is a first line of defence made up of peroxidases and metal chelating proteins which serve as a preventive barrier as they inhibit the formation of ROS and free radicals by capturing metal ions, reducing hydroperoxides, hydrogen peroxide and quenching superoxide and singlet oxygen [6]. A second line of defence is formed by vitamin C and vitamin E which scavenge radicals and so prevent any propagation reactions. The third line of defence of the organism repairs lipids, proteins, sugars and DNA with oxidative damage. This also includes proteases, lipases, DNA repair enzymes and transferases [7]. The antioxidants used could be endogenous such as catalases, which transform H_2_O_2_ to O_2_ and H_2_O, and superoxide dismutases, which convert superoxide radical (O_2_^•−^) to H_2_O_2_ and O_2_, or likewise they could be exogenous, coming from one’s diet. Diet is very important as this provides the antioxidants that intervene in the second line of defence such as vitamin C and E and other antioxidants such as β-carotene, phenols including flavonoids and essential minerals, which participate in the formation of the antioxidant enzymes. Natural antioxidants are found, above all, in vegetables, herbs, berries, spices, tea, coffee and cocoa. For all these reasons, different epidemiological studies have reached the conclusion that consumption of these products, of vegetable origin, are associated with a lower risk of suffering chronic diseases as well as with lower mortality [8,9].

Likewise, antioxidants are a type of additive which is used in the food-processing industry with the aim of preventing an oxidizing deterioration of the lipids as well as preventing loss of nutrition values and the development of odours in the food. The antioxidants additives allowed in food industry may be synthetic or natural, although currently natural antioxidants are more readily acceptable than synthetic antioxidants. The most used synthetic antioxidants in the food processing industry are: butylhydroxyanisole (BHA), butylhydroxytoluene (BHT), propyl gallate (PG) and di-tert-butylhydroquinone (TBHQ), all of which are phenolic synthetic antioxidants. However, since the late XX century the use of synthetic antioxidants additives has become restricted because of their possible toxic and carcinogenic effects [10]. This question has caught the attention of both the scientific community as well as among the general public, and currently there is a lot of interest in developing methods that could provide information and means of isolating both individual antioxidants or those present in extracts coming from different natural sources. Thus, among the positive list of additives permitted by the EU can be found rosemary extract (E392) and different types of tocopherols (E306 an extract rich in tocopherols, E307 α-tocopherol, E308 γ-tocopherol, E309 δ-tocopherol). Given that rose hips are rich in vitamins, especially, vitamin C, as well as phenolic compounds, carotenoids, tocopherol, bioflavonoids, tannins, volatile oils and pectins [11,12,13,14,15], these pseudo-fruits could constitute an alternative source of antioxidants for the food industry as well as serving for therapeutic use.

### 2.2. Presence of Antioxidants in Rose Hips Coming from Different Varieties of Rosa

Rose hips are pseudo-fruits from the plants of the *Rosa* genus in the Rosaceae family. *Rosa* genus contains around 100 species which are widely spread across Europe, the Middle East, Asia and North America [16]. In Europe, the most abundant and most studied is the *Rosa canina* species which is a native shrub [17]. The pseudo-fruits from *Rosa* species have been used both for alimentation and for medicinal purposes thanks to their high level content of bioactive compounds. They are known to have a high level of antioxidant and antimicrobial action [18]. Their antioxidant activity is due to their content in polyphenols, vitamins C, E, B and carotenoids and these compounds may have synergistic effects. Rose hips also have an anti-inflammatory action, as well as anti-diabetic and anticancer effects [19,20,21].

#### 2.2.1. Hydrosoluble Antioxidants

**Figure 1:**
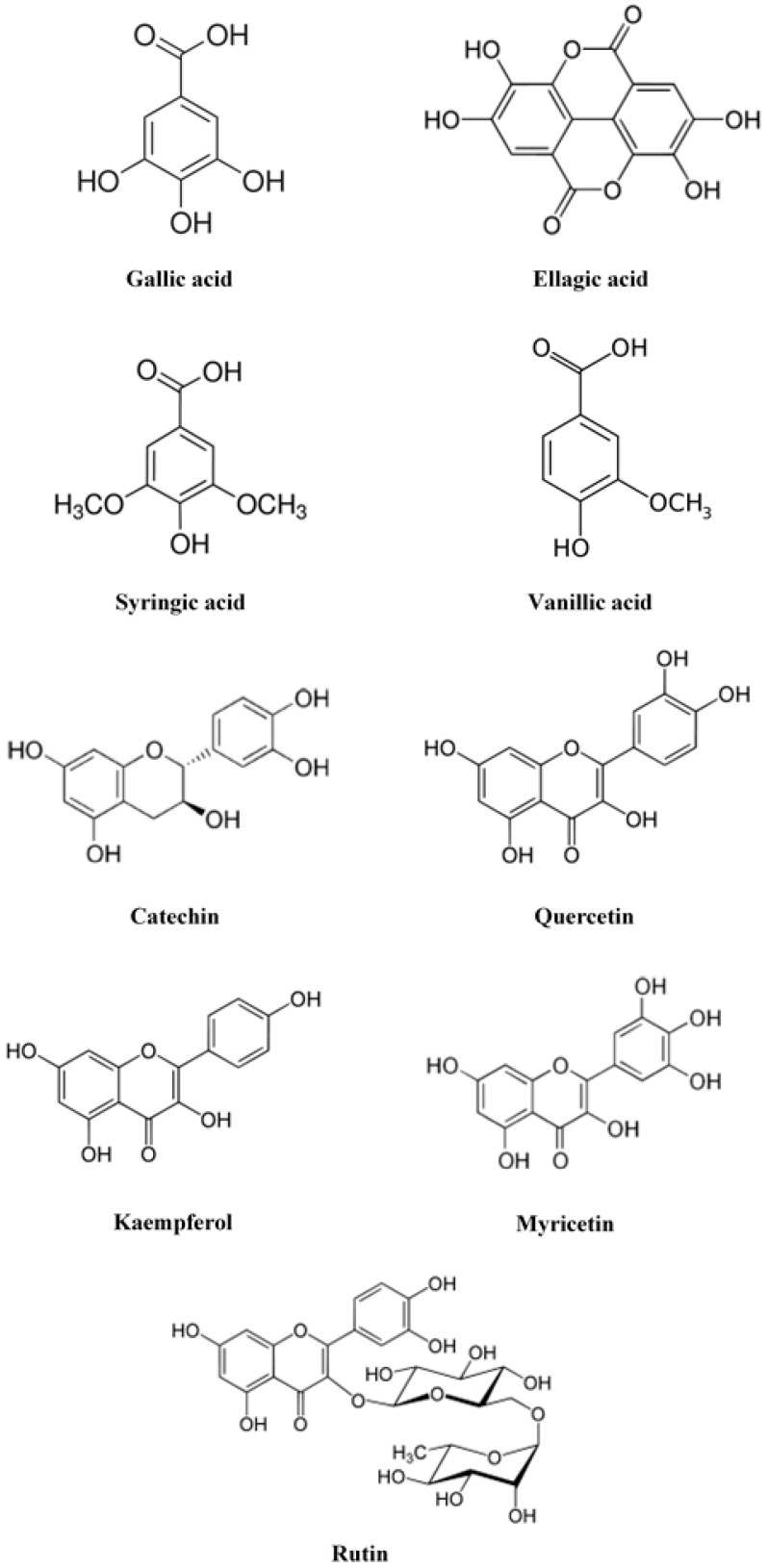
Chemical formulas of hydrosoluble phenolic compounds of rose hips.

Among the hydrosoluble phytochemicals contained in rose hips, phenolic compounds and ascorbic acid stand out [22,23,24,25]. The inclusion of ascorbic acid in diet is important as it provides redox status and produces health benefits. Harrison [26] found a beneficial impact from this vitamin in cognitive decline and Alzheimer´s disease. The concentration of this vitamin in rose hips depends on several factors and has a wide range of concentration. Thus, Czyzowska et al. [27] found 1200 mg/L in *Rosa rugosa* and 600 mg/L in *Rosa canina*. Jiménez et al. [21] found 101 mg of vitamin C/Kg of dry fruit in *Rosa canina* rose hips. This concentration was six times higher than that found by Tumbas et al. [14] in rose hips of *Rosa canina* from Serbia. However, it was lower than those found by Demir et al. [15] in *Rosa canina*, *Rosa dumalis* and *Rosa gallica*, from Turkey and from those found by Türkben et al. [28] in *Rosa canina*, also from Turkey. Phenolic compounds are synthesized in plants through the carbohydrates and are generally produced as a defence mechanism of the plants against pathogens and as a protection against an excess of ultraviolet radiation. These compounds are secondary metabolites of the plants and are characterized by at least one aromatic ring with one or more hydroxyl groups attached. Ellagic acid showed anti-mutagenic and anti-carcinogenic action in different assays both in vivo and in vitro [29]. This compound can act as an antioxidant, and it has also been able to induce apoptosis in carcinogenic cells [30]. Quercetin is the most abundant flavonoid that accumulates in superior plants where it forms glycosides, such as rutin, with a great variety of sugars [31]. Quercetin and its derivative quercetin-3-*O*-glucuronide, inhibit ROS overproduction making chemoprotection of mitochondrial function through antioxidative actions [32]. Tumbas et al. [14] found that *Rosa canina* rose hips from Serbia showed high concentrations of vitamin C, quercetin and ellagic acid. These results coincide with those of Hosni et al. [33] who observed that quercetin and ellagic acid were the most abundant phenolic compounds in Tunisian rose hips. Fujii and Saito [34] found that, among other compounds isolated from *Rosa canina* rose hips, quercetin was a potent melanogenesis inhibitor in B16 mouse melanoma cells. The inhibition of melanogenesis by quercetin was due to the inhibition of both tyrosinase activity and of the protein expression. Conceição de Oliveira et al. [35] reported that a high intake of quercetin was associated with a lower risk of suffering from diabetes type II. Ouerghemmi et al. [36] found that *Rosa canina* extracts includes two phenolic compounds derivatives of kaempferol (kaempferol 3-*O*-glucoside and kaempferol-7-*O*-glucoside). Ercisli et al. [13] studied the chemical composition of the fruits from *Rosa canina*, *Rosa dumalis* subsp. *antalyensis*, *Rosa dumalis* subsp. *boissiere*, *Rosa villosa*, *Rosa pulverulenta* and *Rosa pisiformis*. In this study, it was observed that the highest total phenolic content corresponded to *Rosa canina* (96 mg gallic acid equivalents/g dry weight) and *Rosa pulverulenta* (94 mg gallic acid equivalents/g dry weight), whereas the largest content of ascorbic acid corresponded to *Rosa dumalis* subsp. *boissiere* (943 mg ascorbic acid/100 mL) and to *Rosa pulverulenta* (923 mg ascorbic acid/100 mL). The authors of this study concluded that native rose genotypes are extremely rich in phenols and ascorbic acid and so could be used as functional food or as a food additive. More recently, Demir et al. [15] studied the quantity of phenolic compounds and antioxidant activities of five different rose hip (*Rosa* L.) species including *Rosa canina*, *Rosa dumalis*, *Rosa gallica*, *Rosa dumalis* subsp. *boissiere* and *Rosa hirtissima* grown in Turkey. They found that the greatest amount of phenolic compounds corresponded to *Rosa dumalis* subsp. *boissiere* (52.94 mg gallic acid/g dry weight) and that the rest of them showed very similar concentrations of around 30 mg gallic acid/g dry weight. As for the concentration of individual phenolic compounds, those authors found a high concentration of catechin, procyanidin B2 and epicatechin gallate in *Rosa dumalis* subsp *boissiere*. Aladedunye et al. [37] found that the major phenolic compounds of *Rosa woodsii* fruit extracts were gallic acid, catechin and quercetin, which coincides with the results of Abdel-Hameed et al. [38] in *Rosa damascena* extracts. In Figure 1 are shown the chemical formulas of the most important phenolic compounds present in rose hips.

In addition, some studies have been made comparing the concentration of bioactive compounds in rose hips with that of other vegetables of interest to alimentation and to the food industry. Öztürk et al. [39] compared phenolic acid content in sour cherry juice, rose hips tea, green tea and tomato and found that rose hip tea contained a greater quantity of phenolic acids than the rest of the products. Rose hips contained vanillic acid, p-coumaric acid and ferulic acid in important concentrations, while green tea had high concentrations of syringic acid. Denev et al. [40] studied the concentration of major polyphenol and anthocyanin contents of fruit extract and the antioxidant properties of six fruits: rose hip (*Rosa canina*), rowanberry (*Serbus aucuparia*), hawthorn (*Crataegus monogyna*), chokeberry (*Aronia melanocarpa*), blackcurrant (*Ribes nigrum*), and blueberry (*Vaccinium myrtillus*). They found that rose hips had chlorogenic acid but in lower concentrations than rowanberry and chokeberry, and that the rose fruit showed a high concentration of rutin but no anthocyanins were found. As for the antioxidative properties of these six fruits, rose hip extract showed the highest antioxidant activity via Oxygen Radical Absorbance Capacity (ORAC), Total Peroxyl Radical Trapping Potential (TRAP) and Hydroxyl Radical Averting Capacity (HORAC) assay, while blueberry extract was the most potent inhibitor of lipid peroxidation. These results were similar to those found by Halvorsen et al. [41] who studied the concentration of total antioxidants by the Ferric-Reducing Ability of Plasma (FRAP) assay in different dietary plants (fruits, berries, vegetables, cereals, nuts and pulses), finding that vegetables that contain most antioxidants included members of several families, such as Rosaceae (dog rose, sour cherry, blackberry, strawberry, raspberry).

In some research studies, the composition of other parts of the rose as distinct from the rose hips has also been studied. Guimarăes et al. [42] studied the composition of petals, fertilized flowers, unripe hips, ripening hips and overripe hips from the *Rosa micrantha* from Portugal. These authors found that fertilized flowers showed the highest concentration of total phenolic compounds (527.07 mg GAE/g extract) followed by the petals (424.20 mg GAE/g extract), and that the different hips showed much lower concentrations. A similar study was carried out by Barros et al. [43] in which they made an evaluation of the antioxidant properties (DPPH radical scavenging effects, reducing power, and inhibition of β-carotene, inhibition of lipid peroxidation) of *Rosa canina* hips seed, petals, flowers and galls. They found that galls proved to have the greatest antioxidant activity, ripen hips showed the highest content of tocopherols and β-carotene, unripe hips gave the highest levels of ascorbic acid and petals had the highest concentration of sugars.

#### 2.2.2. Lipid-Soluble Antioxidants

**Figure 2:**
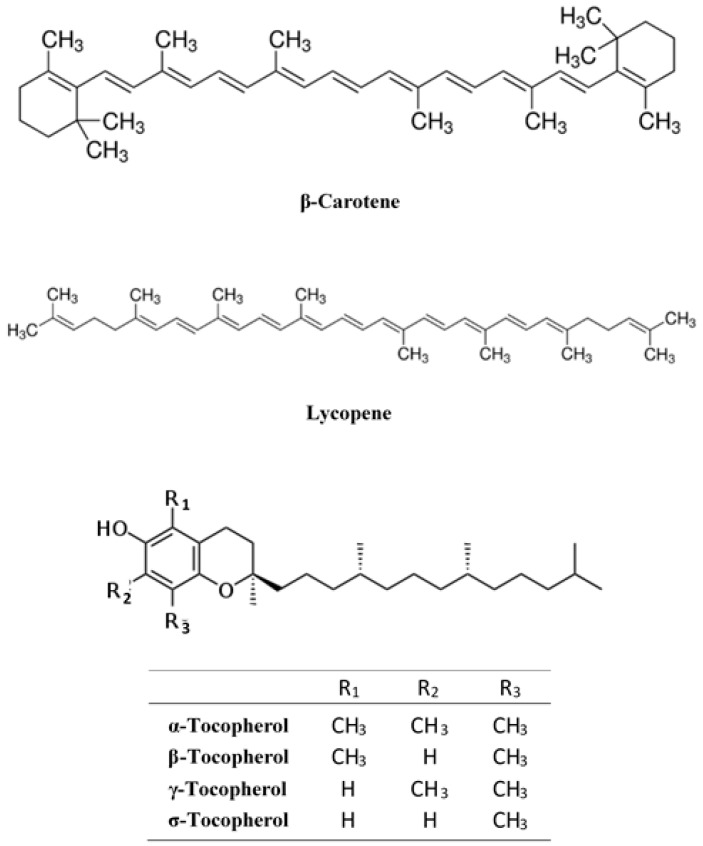
Chemical formulas of lipid-soluble antioxidants of rose hips.

In addition to the hydrosoluble antioxidants, in *Rosa* species there are also lipid-soluble antioxidants such as carotenoids and tocopherols, although these compounds have been less studied and knowledge is more limited than in other bioactive substances. The distinct orange to red colour of rose hips is formed as a result of various carotenoids. The most abundant of these is β-carotene and lycopene, followed by β-cryptoxanthin, rubixanthin, zeaxanthin and lutein [44,45,46]. Carotenoids are very important in the human diet as they act as provitamin A and can prevent certain chronic diseases and even cancer [47]. Tocopherols, α-, β-, γ-, and δ-isomers, and tocotrienols have vitamin E activity and they are compounds with ability to scavenge free radicals, and even tocopherols can be considered as the most important natural antioxidants. Several authors [48,49,50] have shown that these antioxidants have beneficial effects in degenerative diseases such as atherosclerosis, Alzheimer´s disease, cardiovascular disease and certain types of cancer. Fromm et al. [51] studied seed oils from different Rosaceae species such as dessert and cider apples (*Malus domestica* Borkh.), quince (*Cydonia oblonga* Mill.) and rose hip (*Rosa canina* L.). In this study, it was found that qualitative and quantitative composition of tocopherols and carotenoids differed significantly between different genera and also among cultivars of the same species. Total content of tocopherols of the rose seed oils analysed ranged from 597.7 mg/kg from one sample of quince to 1099.9 mg/kg of rose hip. Andersson et al. [46] studied the concentration of tocopherol and tocotrienol in four different species of *Rosa* (*Rosa dumalis*, *Rosa rubiginosa*, *Rosa spinosissima*, *Rosa pimpinellifolia*) and only found α- and γ-tocopherol in the fleshy parts of the rose hips. Tocopherol content and vitamin E activity varied depending on the date of harvesting, species and year, while the fruit ripening had little influence on this activity. In Figure 2 the chemical formulas are shown of the most important lipid-soluble compounds present in rose hips.

### 2.3. Presence of Other Active Compounds in Rose Hips

Although most of the time when speaking of the pseudofruits from the different *Rosa* species, reference is made to their content of vitamin C and antioxidants, there are also other important bioactive compounds in rose hips. For a long time the residue products from rose hips have been used as animal fodder, but nowadays these residue products are gaining in importance as they can be used in cosmetics, pharmacology and in food applications as they contain oil with a high degree of unsaturated lipids. The quantity of oil contained in the seeds depends on the species and ranges from 5 to 18%. In rose hip, seed oil 97% corresponds to linoleic, oleic, palmitic and stearic acid; the remaining 3% is made up of 12 minority fatty acids [52]. Grajzer et al. [53] and Kazaz et al. [54] found that the content of linoleic acid is approximately 40–56% of seed oil, α-linoleic and its derivatives between 20–30% and oleic acid 14–20%. Rose hip seed oil has been used in cosmetics because of its therapeutic effect on skin disorders [55]. In addition, Szentmihalyi et al. [56] compared various methods to extract oil from rose hips, traditional solvent extraction, by ultrasound and microwave sub- and supercritical fluid extraction. Unsaturated fatty acids (oleic, linoleic and linolenic acid) were extracted to a maximum of between 35.9–54.7% using microwave and polyunsaturated fatty acid (linoleic and linolenic acid) were found between 60% and 90% in the recovered oils. 

**Figure 3:**
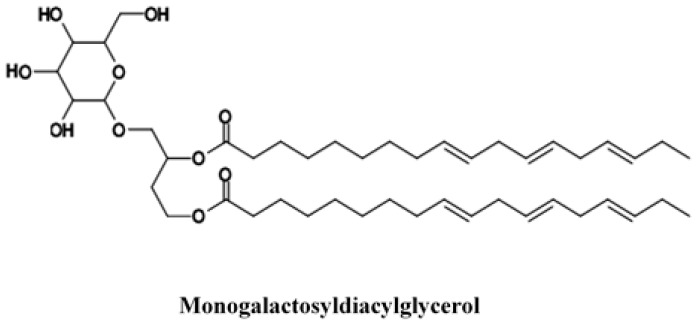
Chemical formula of monogalactosyldiacylglycerol of rose hips.

Likewise, some galactolipids were isolated from *Rosa canina* hips. Galactolipids (monogalactosyldiacylglycerols and digalactosyldiacylglycerol) are compounds that accumulate in the plasma membrane of the plants, and distinguishes them from the lipidic composition of the cell membranes of animals and fungi [57]. Most galactolipids possess fatty acids with high levels of unsaturation. α-linolenic acid can range as high as 90% of the total fatty acids [58]. Lopes et al. [20] found that some galactolipids had an anti-inflammatory action and Maeda et al. [59,60] found that these compounds have anti-tumorigenic roles. Larsen et al. [61] and Cohen [62] isolated the (2*S*)-1,2-di-*O*-[(9*Z*,12*Z*,15*Z*)-octadeca-9,12,15-trienoyl]-3-*O*-β-d-galactopyranosyl glycerol from dried and milled fruits of *Rosa canina* by bioassay-guided fractionation (Figure 3). Christensen [63] isolated the galactolypid 1,2-di-*O*-α-linolenoyl-3-*O*-β-d-galactopyranosyl-sn-glycerol from *Rosa canina* hips. Hou et al. [64] found that bioactive glyceroglycolipid 1,2-di-*O*-α-linolenoyl-3-*O*-β-galactopyranosyl-sn-glycerol (dLGG) that was identified from *C. rabens* was found in vitro and in vivo to be a potent nitric oxide (NO) scavenger. dLGG treatment inhibited both mRNA and protein expression of inducible NO synthase and cyclooxygenase-2 (COX-2) in murine macrophages and inhibited COX-2 gene transcription in 12-*O*-tetradecanoylphorbol-13-acetate (TPA)-treated B16 cells.

There is also some research work where extracts from rose hips have different pharmaceutical actions but the chemical composition of these extracts was not studied. Thus, McCutcheon et al. [65] examined a hundred extracts obtained in methanol from plants in order to determine the antiviral activity against seven virus. It was found that twelve extracts had antiviral activity at non cytotoxic concentrations. The extracts of *Rosa nutkunu* and *Amelunchier alnifoliu*, both members of the Rosaceae, were found to be very active against an enteric coronavirus. Orhan et al. [66] also found in vivo anti-inflammatory and antinociceptive activity from aqueous crude extract, ethanol extract and from different fractions obtained from these earlier extracts of *Rosa canina* L. fruits.

Dogan et al. [67] studied the seed mineral contents of different rose hip species (*Rosa canina* L., *Rosa dumalis* subsp. *boissiere*, *Rosa iberica*, *Rosa heckeliana* subsp. *vanheurckiana* and *Rosa pulverulenta*). Nitrogen, phosphorous and potassium contents of the investigated species varied from 19.039 to 28.076 ppm (N), from 553 to 1080 ppm (P), and from 1142 to 2945 ppm (K). Nickel, lead, and sulphur contents of the investigated species ranged from 13.5 to 190.2 ppm, from 0.35 to 4.35 ppm, from 609.5 to 1152.5 ppm, respectively. Likewise, other minerals present in lesser concentration were also analysed such as sodium, iron, manganese, zinc, copper, magnesium, and calcium. The mineral contents of these species ranked from the highest to lowest as N > K > P > S > Mg > Ca > Na > Fe > Zn > Ni > Mn > Cu > Pb.

### 2.4. Factors Affecting the Concentration of Bioactive Compounds in Rose Hips. Utilization of These Compounds in Agro-Food Industry

Different factors could have an influence on the content of phytochemicals in rose hips. From the results of several studies, it would seem that there is quite a range of variability, especially in the concentration of vitamin C and this could be due to the ascorbic acid instability. Dogan and Kazankaya [68] found that the variability in the content of vitamin C of rose hips depends on the geographical origin of the plant, the species and ecological factors. Roman et al. [25] found that the content of vitamin C of rose hips depends on the quantity of light received by the plant and the oxygen level of the environment. Likewise, many factors could affect the vitamin C content of rose hips, and the fruit freshness level seems to be one of the most important ones. Strålsjö et al. [69] found that dried rose hips had less ascorbic acid content than fresh rose hips. More recently, Baiano and del Nobile [70] concluded that the quantity of vitamin C in vegetables depends on several factors such as the plant variety, soil condition, climate, the length of time since it had been picked and storage. Türkben et al. [28] found that quercetin and (+)-catechin were the main phenolic compounds in *Rosa canina* rose hips but their concentrations were very variable depending on the level of ripeness of the fruits as well as processing conditions and manipulation techniques. Demir et al. [15] found that catechin concentration was notable in different *Rosa* species from Turkey, and it depended on geographical origin and ecological factors (light, temperature, or soil nutrients) just as in the case of vitamin C. Likewise, they also found a high variability in phenolic acids such as vanillic acid, caffeic acid and protocatechuic acid. Cunja et al. [71] found that frost stimulates the formation of β-carotene and lycopene in *Rosa canina* hips but that it reduces the concentration of ascorbic acid and some quercetin glycosides. Likewise, they found that antioxidant ability decreased in frostbitten rose hips. Similarty, studies carried out in our laboratory have compared nutraceutical compounds (vitamin C, neutral phenols and acidic phenols) content of four *Rosa* species rose hips: *Rosa pouzinii*, *Rosa corymbifera*, *Rosa glauca* and *Rosa canina* coming from different geographical zones of Spain with different climatology that can condition fruit characteristics. Results showed a great quantitative variability in ascorbic acid and neutral phenols content, and quantitative and qualitative differences in acidic phenols content, depending on the species. A great correlation between freshness of the rose hips and concentration of neutral polyphenols was found. Significant differences were found in the acidic phenols content among the studied species [72].

Because of their rich composition in bioactive compounds, rose hips have meant an important source of food and medicine for many cultures and currently they are being used in the development of functional foods or to improve the stability and conservation of some foods. Thus, it has been observed that the addition of *Rosa canina* rose hips as a functional ingredient in porcine frankfurters acts as a partial substitute for sodium nitrate. This is important since it means that the concentration of this additive could be lowered [73]. It has also been observed that the phenolic compounds of rose hip are useful for preventing or delaying oxidation in very saturated oils such as canola oil (*Brassica* sp.). Aladedunye et al. [37] studied the composition and antioxidant activity of *Rosa woodsii* extracts, and how this phenolic extract acted on canola oil. On studying the efficiency of these phenolic extracts in protecting canola oil from thermal deterioration, it was found that after 7 days, the formation of lipid hydroperoxides was 2.5 times higher in the control canola oil compared to the fortified sample with *Rosa woodsii* extract. Romero et al. [74] compared the effect of adding α-tocopherol, α-tocotrienol and Moscheta rose (*Rosa rubiginosa*) shell extract to canola oil at high temperature (180 °C) in order to determine how it acted on the stability of this saturated fat. From the results, it was found that the addition of *Rosa rubiginosa* shell extract gave great stability to canola oil. *Rosa rubiginosa* shell extract had 825 μg/mL of α-tocopherol. Utrera et al. [75] examined the effect of frozen storage and the addition of a phenolic-rich dog rose extracts (*Rosa canina* L.) on lipid and protein oxidation of beef patties. Protein oxidation was determined by measuring tryptophan loss and the formation of specific lysine oxidation products: α-aminoadipic semialdehyde (AAS), α-aminoadipic acid (AAA), and Schiff bases. The addition of *Rosa canina* extract inhibited the formation of AAS, AAA and had an antioxidant effect on tryptophan oxidation while it promoted the formation of Schiff bases and incremented the hardness of beef patties.

## 3. Rose Hip and Disease

The use of traditional medicine is still deep ingrained in some cultures even today; therefore thousands of people rely on the therapeutic potential of plants for treating certain diseases in their daily lives [76,77,78,79]. Benefits from of this kind of therapy are not unexpected, since natural products have always been an ample source of new medical compounds [80]. Due to its phytochemical composition, rose hip is an interesting therapeutic option for those disorders which involve oxidative stress and/or a pro-inflammatory status.

### 3.1. Anticancer Activity

Cancer onset and progression is closely related to the intracellular levels of reactive oxygen species (ROS). ROS-induced damage in mitochondrial and nuclear DNA produces mutations responsible for tumour apparition, while lesions in other cellular components such as proteins or lipids contribute to maintaining cancerous phenotype [81]. Moreover, cancer cells are known for their abnormally increased ROS levels, due to the role of these molecules in cell proliferation. In order to avoid oxidative stress damage, antioxidant systems of tumour cells are also increased. Consequently, redox balance in cancer is quite delicate and its disruption triggers cell death. Therefore, many chemotherapy strategies are focused on a redox balance rupture [82,83]. Since medicinal plants are a natural source of antioxidant compounds, many studies highlight the effectiveness of a wide variety of plants against cancer [84]. 

The role of rose hip in cancer treatment has been widely tested among a wide variety of cancer cell lines, and promising results have been obtained in most cases. As the antitumor potential of rose hip is supposed to be a consequence of its phytochemical composition, which may vary attending to the factors described above, quite different results have been obtained by researchers from distinct regions even when they work with the very same rose variety. 

The anticancer properties of *Rosa canina* have been evaluated in various cancer cell lines as shown in Table 1 [14,21,85]. All groups found significant decreases in cell viability after incubating these cancer cell lines with whole rose hip extract or with purified fractions of its most relevant components (vitamin C and neutral and/or acid phenolic compounds). This antiproliferative effect may be related to the antioxidant properties of these extracts. According to Jiménez et al. [21] the incubation of Caco-2 cell line with rose hip fractions resulted in a dramatic decrease in ROS levels. Furthermore, the fractions with the highest antioxidant activity—namely neutral and acid phenolic compounds—were also the ones with the greatest antiproliferative effect. Moreover, Guimarães et al. [85] proved that antiproliferative effects of *Rosa canina* extracts were mainly due to their phenolic content, as non-anthocyanin phenolic compounds enriched extracts showed greater antioxidant activity and provided higher GI50 values than anthocyanins enriched extracts. Some tumours present a reactive oxygen-driven phenotype, which means they use the ROS produced by the Warburg phenomenon as signalling molecules. Patients suffering from these glycolytic tumours, which usually tend to have a poor prognosis, are the most likely candidates to benefit from antioxidant therapy [85,86].

However, Tumbas et al. [14] and Guimarães et al. [85] also noticed that incubation of some rose hip extracts with breast adenocarcinoma (MCF-7) led to an increase in cell viability. They suggest that their content in isoflavone phytoestrogens may stimulate the growth of oestrogen-dependent cells like MCF-7 are [87]. So in conclusion, the therapeutic use of *Rosa canina* may be limited to glycolytic cancer and should be avoided in oestrogen-dependent tumours in order not to promote cancer growth.

Anticancer activity of rose hip is not always linked to its antioxidant properties. Antiproliferative effects of extracts from *Rosa canina* leaves were observed on myeloblastic leukaemia cell model (HL60 cells) and to a lesser extent on myelomonocytic leukaemia model (U937 cells), alone or combined with other plant extracts [88]. Incubation with plant extracts resulted in an upregulation of vitamin D receptor and retinoid X receptor, accompanied by an increase in the transcriptional activity of this receptor complex. Results from Zhamanbayeva et al. [87] suggest that some phytochemicals contained in *Rosa canina* could mimic the mechanism of action of 1,25-Dihydroxyvitamin D3 analogues tested in clinical trials against myeloid leukaemia; these compounds show an intense differentiation-inducing activity [89,90].

Lee et al. [91] found that *Rosa rugosa* extracts inhibited human prostate cancer cell line LNCaP growth due to its anti-histone acetyltransferase activity. Androgen receptor (AR) is a ligand-dependent transcription factor which acts as a key molecule for prostate cancer proliferation. Histone acetylation regulates the transcription of AR-related genes [92], so many anti-prostate cancer drugs are designed to interrupt this process [93]. By inhibiting histone acetyltransferase activity, *Rosa rugosa* extracts repressed the androgen receptor-mediated transcription and as a consequence induced cell death. Moreover, co-administration of *Rosa rugosa* extracts and flutamide—an androgen receptor antagonist commonly used in prostate cancer chemotherapy— resulted in a higher transcription blocking. This synergy effect demonstrates that *Rosa rugosa* extracts may have a promising future in therapy alone or as an adjuvant [91]. Nevertheless, it is still not clear if this anti-histone acetylation activity is due to the action of a particular compound, as yet unidentified, or if it is a result of the combination of various phytochemical compounds. Further research in this field is required. 

*Rosa roxburghii* is another traditional medicinal plant with antitumor properties. Firstly, Liu et al. [94] observed that, in combination with *Fagopyrum cymosum*, extracts from *Rosa roxburghii* induced intrinsic apoptosis on human oesophageal squamous carcinoma (CaEs-17), human gastric carcinoma (SGC-7901) and pulmonary carcinoma (A549). Then, Chen et al. [95] found that crude polysaccharides extracted from *Rosa roxburghii* induced strong cytotoxicity on epithelial ovarian cancer A2780 cell line and reduced cell migration, revealing the potential therapeutic use of *Rosa roxburghii* for the treatment of metastatic ovarian cancer. Regarding the underlying mechanism of action, Chen et al. observed that incubation with crude polysaccharides led to a decreased expression of matrix metalloprotease-9 (MMP-9). MMP-9 is a proteolytic enzyme upregulated in certain tumours, including ovarian cancer, and it is associated with poor prognosis due to its capacity in degrading some components of the extracellular matrix and hence in promoting metastasis [96]. Although the mechanism by which crude polysaccharides from *Rosa roxburghii* reduce MMP-9 expression remains unknown, other studies using polysaccharides extracted from *Inonotus obliquus* revealed that the transcription of MMP-9 was disrupted via RNA transcription factors, as NF-κB signalling pathway was inhibited [97].

### 3.2. Rheumatoid Arthritis

Rheumatoid arthritis (RA) is a chronic systemic disease that affects 22.7% of the adult US population. This condition can strike people of all ages and sexes, but it is most commonly seen between the ages of 20 and 50, and the prevalence is higher in women (23.9%) than in men (18.6%) [98].

RA is a systemic inflammatory disease that usually affects the lining of the joints (synovial membrane), leading to erosions of the cartilage and deformity of bone and joints. In some cases, joint inflammation can be accompanied by generalized systemic symptoms, as well as inflammatory involvement of non-articular organs. Nowadays, the causes of RA remain unknown, and several etiologic mechanisms have been proposed. Although it seems clear that autoimmune disorders play an important role in this disease, other endocrine, metabolic and nutritional factors can also contribute to this pathology [99,100]. 

Nowadays, there is no effective therapy for RA. Different combinations of non-steroidal anti-inflammatory drugs (NSAIDs), disease-modifying anti-rheumatic drugs (DMARDs) and biologic agents are used for the treatment of symptoms. However, new drugs are needed since some patients do not respond successfully and the traditional treatments cause serious side effects in others. As a result, in recent years, different researchers have focused their interest in the study of traditional treatments for RA with the aim of discovering new active compounds present in natural plant extract that could constitute the base for some new drugs for RA treatment (Table 2).

Rose hip, and in particular dog rose (*Rosa canina* L., Rosaceae) have traditionally been used for the prevention and therapy of RA [101]. In particular, rose hip powder has been shown to reduce symptoms associated with rheumatoid inflammation in clinical trials [62,102,103,104]. This effect is related to its high content in anti-inflammatory molecules, such as the galactolipid GOPO (1,2-di-*O*-α-linolenoyl-3-*O*-β-d-galactopyranosyl-sn-glycerol) [60], which can reduce chemotaxis of peripheral blood polymorphonuclear leukocytes, neutrophils and monocytes, as well as diminish the levels of C-reactive protein (CRP), a protein produced in the liver in response to inflammation [102,105]. Moreover, rose hips have been shown to reduce the production of several key pro-inflammatory cytokines (TNF-α, IL-1β, IL-6, (IFN)-γ, IL-12) and chemokines CCL5 (RANTES), IP-10 (CXCL10) [106,107,108].

On the other hand, several fatty acids present in rose hips, such as triterpenoic acids, ursolic acid, oleanolic acid and betulinic acid among others, have been shown to inhibit cyclooxygenase (COX) 1 and 2 activity. COX expression facilitates the invasion of synovial fibroblasts in joints of RA patients and the accumulation of advanced glycation end products. The inhibition of COX contributes towards reducing inflammation in RA patients, and can be responsible for a part of the clinically observed effect [109,110].

The inflammatory process triggered by RA has been also associated with an increased generation of reactive oxygen and nitrogen species (ROS/RNS), which contribute to tissue damage. In consequence, antioxidant nutrients such as vitamin C, vitamin E, carotenoids, polyphenols and antioxidant enzymes could play a significant role in the protection against the damaging effects of ROS/RNS produced in RA [100]. It has been shown that lipophilic extracts and polyphenols from rose hips reduce the production of ROS and inhibit NO release from macrophages [107,111]. Moreover, total flavonoids from rose hips protect against cell apoptosis, DNA and mitochondrial H_2_O_2_-induced damage as well as amyloid beta peptide-induced oxidative injury [21,112,113].

Another alteration commonly found in RA patients consists of a dysregulation of Nuclear Factor (NF-κB) signaling pathway. NF-κB regulates a vast variety of biological processes including immunity, inflammation, and apoptosis. In RA, the imbalanced production of pro-inflammatory mediators and oxidative damage promote a chronic activation of NF-κB pathway, which results in inflammation, hyperplasia and tissue destruction [114]. As a result, the inhibition of NF-κB constitutes a potential target for RA. In this context, several members from the *Rosa* spp. have shown inhibitory actions on NF-κB related inflammatory responses. For instance, phytosterols present in *Rosa* spp., such as, β-sitosterol, attenuates the phosphorylation of NF-κB in TNF-α-stimulated human endothelial cells [115]. Phenolic compounds, such as gallic acid, re-establishes the association of IκBα with NF-κB, hampering the capacity of NF-κB to bind DNA [116], while astragalin and tormentic acid inhibit IkBα phosphorylation and degradation as well as NF-κB nuclear translocation [111,117].

Besides inflammation, RA patients suffer from abnormal bone destruction caused by an abnormal activation of osteoclast. Osteoclast differentiation is dependent on cytokines and membrane-bound factors expressed by T cells. Receptor activator of NF-κB ligand (RANKL) is one of the major osteoclastogenic cytokines. When the ligand binds to RANK, IκBα kinase (IKK), NF-κB, and nuclear factor of activated T cells, cytoplasm 1 (NFATc1) are sequentially activated, leading to osteoclastogenesis [118]. Aqueous extract of rose hip has been found to inhibit the activation of NF-κB mediated by RANKL and slow down the RANKL-induced osteoclastogenesis, and hence it could constitute a bioactive molecule against bone destruction in RA [119].

Consequently, thanks to its composition in bioactive molecules, different members of *Rosa* genus have proved to have analgesic and anti-inflammatory actions in different pain and inflammatory animal models of RA [120].

### 3.3. Osteoporosis

Osteoporosis is a common disease characterized by a decrease in bone mass and strength, as well as an alteration of bone microarchitecture. The main consequence of this disease is an increase in the risk of developing bone fractures especially of the hip and vertebrae [121].

In bone, ROS play a dual role. Under physiological conditions, the production of ROS by osteoclasts facilitates destruction of calcified tissue, assisting in bone remodelling. Additionally, in bone fractures, the interaction between collagen strand and the mineral phase generates large amounts of ROS which may facilitate bone healing [122]. However, an enhanced osteoclastic activity may increase the superoxide anion generation and inhibit superoxide dismutase and glutathione peroxidase activities, triggering oxidative stress [123]. This abnormal increase of oxidants can over-activate different proteolytic enzymes, such as neutrophil elastases or metal proteinases, promoting the oxidative damage of bone extracellular matrix, with a consequent alteration of cell metabolism and viability [122]. Therefore, antioxidant supplementation can contribute to restoring Bone Mineral Density (BMD) by regulating the levels of oxidative stress. In a ovariectomized rat model of post-menopausal osteoporosis, antioxidants contained in blueberries reduced femoral mRNA levels of alkaline phosphatase, collagen type I, tartrate-resistant acid phosphatase as well as the levels of serum mRNA of osteocalcin. This treatment also reduced loss of whole-body BMD and prevented the loss of tibial and femoral BMD [124]. Extracts of *Rosa* spp. has a similar phytochemical profile to blueberries, characterized by a high content in antioxidants and consequently, they could also play an important role in preventing osteoporosis [125].

On the other hand, antioxidants play a key role in the regulation of osteoblast differentiation. For instance, ascorbic acid (AA), is a cofactor required for hydroxylation and secretion of procollagen, and for the formation of the stable-triple-helical collagen, molecule needed for the correct growing and maturation of both connective and bone tissue [126]. Moreover, AA can potentially bind putative antioxidant-responsive elements (ARE) contained in many of osteoblastic and osteocitic genes, such as osterix, and induce their expression, stimulating Bone Marrow Mesenchymal Stem Cells (BMMSC) to differentiate into osteoblast [127]. However, no study has yet showed this activity by *Rosa* spp. extracts.

Several studies have demonstrated that natural antioxidant supplementation including polyphenols reduce bone loss caused by oxidative stress [128]. As a result, *Rosa canina*, thanks to its high content in antioxidants, constitute a potential treatment for osteoporosis by reducing the damage caused by an excess of ROS in bone tissue, and by increasing bone formation through the synthesis of collagen matrix and the stimulation osteoblast differentiation [124,125].

### 3.4. Diabetes

Diabetes mellitus is a group of physiological dysfunctions characterized by hyperglycemia resulting directly from inadequate insulin secretion, insulin resistance, or excessive glucagon secretion. Diabetes mellitus has been classified in two types: Type 1 diabetes (T1D)—an autoimmune disorder that provoke the destruction of pancreatic β-cells and Type 2 diabetes (T2D)—an alteration in glucose regulation due to a combination of dysfunctional pancreatic β cells and insulin resistance [129]. Nowadays, diabetes mellitus is the most common endocrine disorder and its incidence is rising year by year [130].

Several studies have demonstrated that the overproduction of ROS has a key role in the severity of diabetes complications. In diabetic patients, hyperglycemia induces overproduction of superoxide by the mitochondrial electron-transport chain, which increases hexosamine pathway flux, enhances the formation of advanced glycosylation end products and activates protein kinase C. Moreover, ROS also enhance nitric oxide generation favoring peroxynitrite, a strong oxidant which damages DNA. DNA damage activates the nuclear enzyme poly(ADP-ribose) polymerase, and consequently reduces the levels of NAD+, a subtract for poly(ADP-ribose) polymerase. Depletion of NAD+ slows the rate of glycolysis, electron transport, and ATP formation, and produces an ADP-ribosylation of the GAPDH. These processes result in acute endothelial dysfunction that also contributes to the development of diabetic complications [131]. Several antidiabetic drugs, as well as the natural antioxidant vitamin E, have been administrated in medical practices. However, prevention of secondary complications turned out to be insufficient, and in some cases, side effects associated with the treatment prevented their use. In consequence, new therapeutic approaches are needed [130] [132]. In this context rose hip, due to its high content in antioxidants, could constitute a potential adjuvant therapy for diabetes mellitus.

One of the main causes of diabetes mellitus is the deficiency of pancreatic β-cell viability and performance. *Rosa canina* extract with the concentration of 0.001 mg/mL significantly increased proliferation of βTC6 cell line compared with control cells. These results were supported by Orhan et al. [133], who found that R-H_2_O fraction of *Rosa canina* fruits reduced blood glucose level significantly in streptozotocin (STZ)-induced diabetic rats (50–62%) and demonstrated the hypoglycemic effect of *Rosa canina* in an animal model of diabetes mellitus. Moreover, histopathological studies of organs from STZ-induced diabetic rats revealed that low doses of *Rosa canina* extract (250 mg/kg body weight) significantly increased the number of islets in comparison with diabetic rats of the control group and improved the histology of necrotic islets of pancreas [134]. This effect was not associated with the phenolic content *Rosa canina*—which is mainly contained in ethanol and methanol extracts—but with different bioactive compounds such as vitamin B and C, carotene, organic acids, flavonoids, tannins, monosaccharides, oligosaccharides, and pectins contained in the polar extract [135]. For their part, Can et al. [136] demonstrated that the aqueous and ethanol extracts of *Rosa canina* fruits had no hypoglycemic activity in normal rabbits. However, despite the potential of rose hip extract on improvement of cell proliferation, it does not avoid DNA damage and consequently it is not able to prevent the reduction of β-cell viability associated with the glucotoxicity seen in diabetes mellitus type 2 [137].

In diabetes, hyperglycaemia is also associated with more glycogenesis and less glycogen production, so reduction of glucose uptake in liver could ameliorate the complications of diabetes. Rose hip extract was not able to modify glucose uptake in the hepatic cell line HepG2. Nevertheless, in high fat diet models, rose hip extracts down regulated the hepatic lipogenic program, resulting in a reduction of >50% of the hepatic lipids, and a consequent improvement in insulin sensibility and glucose toleration, as well as a reduction of plasma cholesterol levels. The mechanisms underlying these effects remain unknown and further investigations are needed [46].

Another therapeutic approach for diabetes type 2 consists in delaying the production or absorption of glucose by inhibiting carbohydrate hydrolysing enzymes such as α-amylase and α-glucosidase to decrease postprandial hyperglycaemia [138]. In vitro models of glucose absorption revealed that rose hip extracts were not able to alter glucose diffusion [137]. Nevertheless, rose hip extracts, and garlic acid contained in them among other molecules, can inhibit both enzymes. The inhibition of α-amylase is lower than the repression on α-glucosidase activity. As a result, sugars can be degraded into smaller carbohydrates passing through gastrointestinal tract without undesirable microbial fermentation, but, as α-glucosidase activity is highly repressed, rapid glucose release to bloodstream is prevented, contributing to the regulation of blood sugar level [9]. The molecules responsible for these effects have not been identified and further studies are needed in this field. 

### 3.5. Hyperlipidaemia

Hyperlipidaemia is a condition consisting of elevated plasma cholesterol and/or triglyceride levels. Hyperlipidaemia is associated with an increased risk of cardiovascular disease (CVD), high risk of developing premature coronary artery disease (CAD), hypercoagulability, hyperinsulinemia, insulin resistance, and glucose intolerance pancreatitis [139].

Niominya et al. [140] demonstrated that 80% aqueous acetone extracts from the fruit (50 mg/kg/day and seeds (12.5 and 25 mg/kg/day) of *Rosa canina* significantly reduced plasma triglyceride (TG) and free fatty acid (FFA) levels after 14 days of treatment in mice. The main bioactive compound of this extract is *trans*-tiliroside, whose administration significantly reduced liver TG levels, and tended to diminish FFA levels, but not significantly. Niominya et al. also found that *trans*-tiliroside increased the expression of peroxisome proliferator-activated receptor α (PPAR-α) mRNA levels in liver tissue 24 h after single oral administration [140]. PPAR-α is a transcription factor that induce the expression of PPAR-acyl-CoA oxidase gene and therefore lead to reduced FA substrate for TG synthesis [141]. Although more profound studies should be made, these results suggest that the anti-lypidemic effect of *Rosa canina* could be based on its capacity to promote the lipid metabolism [140].

These results were also confirmed by Taghizadeh et al. (2016) who found that the administration of *Rosa canina* extract in STZ-induced diabetic rats at a dose of 250 mg/kg body weight significantly decreased the levels of serum triglycerides whereas serum cholesterol, low-density lipoprotein, and high-density lipoprotein levels were not modified [134].

On the other hand, GC-MS analysis of seed oil from several species of rose hip revealed a high content in polyunsaturated fatty acid ω-3 and ω-6 (45.38–54.58% linoleic acid (ω-6), 13.67–24.75% alpha linolenic acid (ω-3), 20.83% oleic acid (ω-9), 12.97% palmitic acid, 8.54% stearic acid, and 1.99% arachidonic acid [142]. The results obtained revealed that the oil from the seeds of rose hip contains polyunsaturated fatty acids ω-3 and ω-6 which decrease TG levels. The mechanisms underlying this reduction of plasma TG levels are complex. On the one hand, polyunsaturated fatty acid, such as the ones contained in rose hip extracts, prevent the binding of the LXR/RR heterodimer to the regulatory regions LXREs contained in SREBP-1c promoter, and consequently, reduce the expression of SREBP-1c [143]. SREBP-1c is a transcription factor with a key role in the control of hepatic lipogenesis. Suppression of SREBP-1c expression results in a reduction of acetyl-CoA carboxylase and FA synthase, resulting in a decrease in FA synthesis. Moreover, polyunsaturated fatty acids decrease the activity of key enzymes in TG biosynthesis, such as phosphatidic acid phosphohydrolase or diacylglycerol (DG) acyltransferase that catalyses phosphatidate to DG and DG to TG, respectively. Secondary to a decrease in TG synthesis, polyunsaturated fatty acid can also inhibit the synthesis and release of decrease hepatic very low-density lipoprotein (VLDL)-TG. On the other hand, polyunsaturated fatty acids can suppress the expression of hepatic lipase and Apo CIII and increase Apo CII and VLDL-receptor [144].

### 3.6. Obesity

Obesity rates have experienced such a great increase over these last years that it is now considered to be global epidemic. Changes in diet and lifestyle are the major causes for this increase in incidence and the consequences of obesity include a wide range of disorders, from diabetes to cancer [145,146,147]. In this context, prevention of obesity would contribute towards avoiding all its related diseases, with a resulting improvement in the quality-of-life for many thousands of people worldwide. Obesity can be basically classified as an inflammatory process, since adipocytes dysfunction results in an increased production of pro-inflammatory cytokines [148]. Due to the anti-inflammatory properties of some phytochemicals contained in medicinal plants, it is reasonable to suppose that they could play a therapeutic role in obesity [117,149].

Focusing on the potential benefits of rose hip for obesity prevention, Ninomiya et al. [140] reported the anti-obesity properties of *trans*-tiliroside (kaempferol 3-*O*-(6′′-*p*-coumaroyl)-β-glucoside), a glycosidic flavonoid contained in *Rosa canina*. Administration of rose hip extracts of *trans*-tiliroside alone to rat models resulted in a reduction in overweight and liver fat accumulation. Furthermore, lipid metabolism was found to increase after *trans*-tiliroside administration, since peroxisome proliferator-activated receptor α (PPARα) was overexpressed. PPARα decreases lipid levels, as its activation promotes fatty acids oxidation, and the use of PPARα agonists ameliorate obesity-related side effects [150]. 

Nagatomo et al. [151] delved into the effect of *Rosa canina trans*-tiliroside in obesity. They observed that both *Rosa canina* extract and *trans*-tiliroside were able to prevent lipid accumulation in differentiated adipocytes cell model. On mouse models, both conditions led to a decrease in body weight. Finally, they found a significant decrease in PPARγ expression after *Rosa canina* extract administration. As PPARα, PPARγ is implicated in lipid homeostasis, as well as being involved in adipogenesis and in insulin sensitivity control [150]. Taken together with the previous discovers of Ninomiya et al. [140], results from Nagatomo et al. [151] show that *trans*-tiliroside anti-obese effects are related to an increase in lipid metabolism linked to a decrease in adipogenesis. 

Arising from these findings, Nagamoto et al. [152] investigated the effects of daily intake of rose hip extracts on pre-obese volunteers. They observed that this supplementation contributed to reducing the levels of abdominal visceral fat without any undesirable side effects. Thus, they confirmed the previous findings in animal models and proposed *Rosa canina* as a promising candidate for anti-obese therapies. 

### 3.7. Renal Disturbances

Acute kidney injury (AKI) is a disorder characterized by a disruption of regular kidney function that causes renal failure. Since two of the major causes of AKI are oxidative stress and inflammation [153], the previously described antioxidant and anti-inflammatory properties of rose hip make it an interesting therapeutic option for AKI. Using a rat model, Ashtiyani et al. [154] investigated the effect of *Rosa canina* oral administration in AKI induction, and observed a decrease in oxidative stress that correlated well with the observed reduction of renal damage. 

Similar results were obtained by Zhao et al. [155] using flavonoids extracted from *Rosa laevigata*. In this particular case, they observed that incubation with *Rosa laevigata* flavonoids reduced ROS levels in renal tubular duct epithelial cells from rat (NRK-52E), which correlated with a protective effect on kidney injury. This decrease in ROS levels seems to be related to an increase in certain redox enzymes including superoxide dismutase or glutathione peroxidase. Moreover, anti-inflammatory properties of flavonoids were also involved in attenuation of AKI, since incubation with them resulted in a decrease in the expression of NF-κB related genes. Finally, they tested the effect of *Rosa laevigata* flavonoids on rat models and confirmed their nephroprotective effect. 

Diabetic nephropathy (DN) is one of the most common diabetes-associated injuries and it is related to an overproduction of reactive oxygen species [156]. Encouraged by this fact, Zhou et al. [157] studied the effect of *Rosa laevigata* administration to diabetic rats. Treatment with *Rosa laevigata* reduced reactive oxygen species levels via increasing superoxide dismutase levels-like Zhao et al. [155] observed-, which eventually resulted in an ameliorative effect from renal injury. Once again, the expression of NF-κB related genes decreased after incubation with *Rosa laevigata*, showing unambiguously the anti-inflammatory potential of this plant. 

Calcium oxalate nephrolithiasis is a polygenic disorder whose incidence is rising worldwide every day. Kidney stones are usually removed using extracorporeal shock wave lithotripsy, although this technique may involve undesirable side effects. Currently, there is an important lack of drugs for the prevention and treatment of this disease. Researchers have consequently focused on developing prevention strategies in order to avoid calcium oxalate stones formation [158,159]. Tayefi-Nasrabadi et al. [160] used a nephrolithiasic rat model in order to test the effect of *Rosa canina* extracts on calcium oxalate stones formation. They observed a decrease in calcium oxalate content in treated rats as well as a drop in number of calcium oxalate calculi. This effect is supposed to be result of the antioxidant activity of *Rosa canina*, since they found a significant reduction in lipid peroxidation in kidney, one of the main risk factor for nephrolithiasis. In conclusion, all data collected by Tayefi-Nasrabadi et al. [160] show the potential therapeutic role of *Rosa canina* in prevention and even treatment of nephrolithiasis.

### 3.8. Hepatotoxicity

Liver plays a key role in metabolism, detoxification and energy storage in animals. Due to its functions, this organ is highly exposed to multiple xenobiotics and suffers from high levels of oxidative stress, which can cause deleterious effects to its function [161]. Despite its importance, hepatic diseases are lacking in effective treatments nowadays, and herbal medicines are one of the main options for prevention and therapy [162].

Many hepatic injuries are characterized by lipid peroxidation, generation of free radicals and reduction of the activity of antioxidant enzymes. In clinic, this hepatic injury is turned into increased serum levels of ALT, AST and ALP proteins and reduction of ALB and TB serum protein levels. 

In vivo studies revealed that treatment with the fruit extract of *Rosa canina* at 500 and 750 mg/kg doses restored the levels of these marker enzymes in a model of hepatic injury induced by CCl_4_. Moreover, *Rosa canina* extracts attenuated the histopathological alteration produced by this compound. It is thought that the mechanism of action of *Rosa canina* in liver diseases is based on its content in antioxidants and phenolic compounds which can reduce the peroxidation of unsaturated fatty acids induced by CCl_4_, preventing the damage of cell membranes [163]. 

### 3.9. Neuroprotective Activity

Alzheimer is an age-related neurodegenerative disease influenced by genetic as well as environmental factors which mainly affects the elderly population in developed countries. Its onset is characterized by progressive memory loss accompanied by several changes in behaviour and cognition. With regard to the molecular insights into the pathogenesis of Alzheimer, one of the best characterized abnormalities from Alzheimer is the extracellular accumulation of amyloid β and the formation of neurofibrillary tangles composed by high amounts of hyperphosphorylated tau protein; this eventually leads to on neural loss and gliosis [164]. ROS imbalance is supposed to play a relevant role in Alzheimer development, since abnormal mitochondrial function and consequent oxidative stress have been observed in Alzheimer disease. In this context, antioxidant therapy may be a promising tool for treating this disorder [165]. 

At this moment, *Rosa damascena* is one of the most promising medicinal plants for Alzheimer treatment. Esfandiary et al. [166] supplemented Alzheimer-induced rats with *Rosa damascena* extracts and they found an improvement in spatial and long-term memory. The antioxidant properties of flavonoids content in these rose hips are the most likely elements responsible for their neuroprotective effect, as they are supposed to inhibit the synthesis of amyloid β. Concretely, the flavonoid myricetin has shown the most relevant anti-Alzheimer potential, since it is able to avoid amyloid β formation and has certain tau-antagonist activity. Moreover, its antioxidant properties seem to act as a protection against neuronal damage [167,168].

In quite a similar way, oxidative stress is linked to other neurodegenerative diseases such as epilepsy. The fact that lipid peroxidation has shown to have increased in epileptic patients, whereas vitamins A and C plasma levels were found to be significantly decreased, leads researchers to suppose that antioxidant therapy may be helpful [169]. In fact, flavonoid supplementation has been confirmed as a potential therapeutic tool for epileptogenesis due to its intrinsic antioxidant properties previously discussed. In addition, some flavonoids are able to bind the GABAA-Cl channel complex, acting as anticonvulsive agents [170].

The anticonvulsant and neuroprotective effects from *Rosa damascena* were resumed by Homayoun et al. [171] using a rat model. *Rosa damascena* extracts administration ameliorated convulsions and also reduced the formation of dark neurons, consequently improving memory impairments. Dark neurons are commonly observed in brain-related diseases, and their apparition seems to be linked to oxidative stress [172], so the neuroprotective effect of *Rosa damascena* may be caused by its antioxidant activity. 

Aromatherapy has been proposed as adjuvant therapy for the management of psychiatric disorders including anxiety or depression. Benefits of aromatherapy are due to the chemical composition of essential oils, which contain volatile organic compounds capable of stimulating different central nerve system areas. This stimulation leads to a release of neurotransmitters, for example serotonin, responsible for the observed pro-relaxing effects [173]. Along these lines, relaxing properties *of Rosa damascena* oil have been reported [174] and consequently might have a promising future in psychiatric disorders treatment. 

Mostly of the depression-induced brain damages are related to an overproduction of ROS in the brain that triggers cell death. Therefore, high antioxidant content of rose hip makes it a potential and powerful help for treatment of depression. Nazıroğlu et al. [175] found that *Rosa damascena* inhalation reduced lipid peroxidation levels in depressed rat cerebral cortex, proving the potential of rose hip in the management of depression.

### 3.10. Skin Disorders and Aging

One of the most common skin disorders is atopic dermatitis (AD), a chronic inflammatory disorder that mainly affects children. Treatments for skin lesions associated with this disease are focused on a suppressed inflammatory response, but most therapies are just temporal because of the side effects associated to long time exposure [176]. In order to improve quality-of-life for thousands of infants worldwide, new anti-AD drugs are needed, in order to find a definitive solution to this problem without deleterious effects. 

Topical application of extracts from *Rosa multiflora* root extracts improved AD-like injury in mice [177]. Anti-AD activity of *Rosa multiflora* seems to be related to its anti-inflammatory properties, since application of extracts reduced mRNA levels of inflammatory mediators cyclooxygenase 2 (COX-2) and inducible Nitric Oxide Synthase (iNOS). Moreover, rose hip treatment avoided allergic response derived from AD by decreasing blood eosinophil ratio and plasmatic IgE levels. Finally, *Rosa multiflora* showed an interesting regulatory effect of Th2-immune response, as serum levels of Th2 were significantly decreased after treatment. Nowadays, the most effective anti-AD drugs are those focused on suppressing Th2-polarized immune system [176], so these findings suggest a promising future for *Rosa multiflora* in atopic dermatitis treatment. Of the different components that can be found in *Rosa multiflora* roots, the most likely candidate to act against AD is the condensed tannin RM-3. RM-3 is the most abundant phytochemical in *Rosa multiflora* root extract, and Park et al. [177] proved that this isolated tannin reproduced the effects previously observed for the whole extract.

Skin aging is due to the majority contribution of two factors: on the one hand, chronic aging associated with time, and on the other hand, the commonly so called photoaging caused by UV exposition. The combination of both conditions results in a loss of functionality of skin barrier and in the acquisition of unsightly features such as dryness, wrinkles and spots [178]. The previously discussed antioxidant and anti-inflammatory properties of rose hip make it a promising aid in reducing these skin aging signs.

Fuji et al. [34] investigated the effect of quercetin isolated from *Rosa canina* on melanogenesis in B16 mouse melanoma cells. Melanin is responsible for the pigmentation of human skin, hair and eyes, but its excessive biosynthesis leads to skin disorders like age spots or melanoma [179]. The key enzyme in this process is tyrosinase, and quercetin from *Rosa canina* was found to be capable of inhibiting its activity and consequently reduced melanin content of mouse melanoma cells. Interestingly, this reduction in melanin content was not related to a decrease in cell viability, a key point for their potential application in the cosmetic industry. Later, the oral administration of rose hip extracts to brown guinea pigs decreased skin pigmentation, proving their melanogenesis inhibitor effects in vivo and suggesting the potential use of *Rosa canina* as a skin-lightening agent in cosmetic [180]. 

Phetcharat et al. [181] tested the activity of a commercially available *Rosa canina* powder upon aging-related effects. They carried out a randomized, double-blind controlled clinical trial on healthy middle-aged male and female volunteers and studied the effect of rose hip powder on (1) skin wrinkles and (2) red blood cells longevity. Regarding the first part of their study, rose hip powder reduced the depths of crow’s-feet wrinkles, increased moisture content of forehead and improved skin elasticity. On the other hand, *Rosa canina* powder reduced red cell membrane disintegration and, as a result, increased cell longevity. The anti-aging effects of *Rosa canina* are related to its antioxidant properties, as certain phytochemicals are able to scavenge reactive oxygen species produced by UV-radiation and so reduce skin damage. Vitamin C might have a dual role in skin protection since as well as its antioxidant effect; it is directly involved in skin and collagen formation. Moreover, the anti-inflammatory effect of some components of *Rosa canina* is also related to protection from UV-induced inflammation and damage. Finally, antioxidant compounds and poly-unsaturated fatty acids are the major contributors to *Rosa canina* effects on increasing red cell longevity, as both prevent cell membrane damage.

Part of the anti-aging effect of rose hip is due to its activity as PPAR-α agonist. PPAR-α is involved in inflammatory processes, since its activation leads to an inhibition of pro-inflammatory genes expression like matrix metalloproteases (MMP). The normal function of MMP has been described earlier, which consists of degrading components of extracellular matrix [96]. Consequently, MMPs also play a key role in photoaging, whose synthesis is increased after UV exposure [182]. Results from Jeon et al. [183] agreed with this hypothesis: they observed that *Rosa multiflora* extracts act as PPAR-α agonist which induced a decrease in MPP expression levels, ameliorating photoaging-related effects on murine skin.

Oxidative stress is not the only thing responsible for skin damage, as psychological stress induces deleterious effects on skin barrier, as well. Some of the reported effects are a delay in skin barrier function recovery, an increase in the number and activity of natural killer cells and an increase in the release of pro-inflammatory cytokines such as TNF-α or IL-β [184]. Evidences of the anxiolytic effect of rose hip have been previously discussed [174], but these data provide a new perspective on the beneficial effect of *Rosa* species for skin health. Relaxing properties of *Rosa damascena* oil inhalation resulted in a decrease in transepidermial water loss, a common skin damage feature induced by chronic stress [185].

### 3.11. Diarrhoeal

Diarrhoea is defined as a situation in which an adult daily stool exceeds 200 g and contains 60–95% water [186] and it constitutes one of the leading causes of morbidity and mortality in developing countries [187], where people still rely on healing practices and medicinal plants. As a result, despite the advances of Modern Medicine, the World Health Organization encouraged studies for the treatment and prevention of diarrheal diseases depending on traditional medical practices.

In vivo studies showed that the methanol extract *Rosa canina* had a significant antidiarrhoeal activity mediated by an antisecretory mechanism that induce a fluid accumulation comparable to that obtained with the standard drug, diphenoxylate. Furthermore, *Rosa canina* extract significantly reduced intestinal transit, thereby increasing the absorption of water and electrolytes [188].

The molecular mechanism underlying the antidiarrheal activity of *Rosa canina* extract could be based on the inhibition of acetylcholine and histamine through some secondary metabolites present in the leaf extract, such as flavonoids [187,188,189,190], and saponins [188]. 

Although further studies are needed to identify the active principle responsible for the anti-diarrhoeal activity of *Rosa canina*, its use could constitute a potential therapy for this intestinal alteration. 

### 3.12. Anti-Ulcerogenic Effect

Peptic ulcer disease is an increasing incidence disorder that presents epigastric pain among other common symptoms such as bloating or nausea. It is considered to be a multifactorial disorder which finally derives in a loss of the protective mucosal barrier. Although there are various risk factors associated with the onset of ulcer disease, most of the cases are caused by *Helicobacter pylori* infection and so treatment is focused on antibiotic therapy [189]. 

Anti-ulcerogenic effect from *Rosa canina* extracts was first discovered by Gürbüz et al. [190]. By inducing ulcerogenesis on rat models, they observed that *Rosa canina* were able to totally prevent ulcer formation. However, they did not propose any mechanism of action that explains the observed effects. Lattanzio et al. [191] went deeper in their studies and observed that treatment with *Rosa canina* prevented gastric mucosa erosion and avoided haemorrhagic ulcer formation. They propose that the antioxidant activity of rose hip extracts—which was conserved even at gastric acid pH—was responsible for the results obtained, since lipid peroxidation and ROS production are highly involved in gastric damage.

### 3.13. Antimicrobial Effect

Infectious diseases have a great impact on world health and the worrying and increasing rates of antibiotic resistance intensify pharmaceutical industry research for new drugs in order to solve this emergency situation [192]. In this context, plant phenolic compounds are an interesting source of new antibiotics, especially against Gram-positive bacteria. Regarding their mechanism of action, due the wide variety of structures they possess, each compound interacts with a different cell component. Some of the best characterized phenolic-induced antibacterial effects include increase of membrane permeabilisation, nutrients deprivation and extracellular enzymes inhibition [193]. 

The antibacterial effect of some rose hip species was roughly investigated in Gram positive and Gram negative bacteria strains [192,193,194,195,196], as shown in Table 3, Table 4 and Table 5. Interestingly, these tests confirmed the presence of antimicrobial compounds in different parts of the plant, as they investigated the effects of flowers [193], seeds [194] and fruits [193,195]. Results from Olech et al. [195] focus on the correlation between antioxidant capacity from rose hip extracts—due to their phenolic content—and their antibacterial potential. The mechanism of action they propose is an energy deprivation caused by a hyperacidification of plasma membrane interface that disrupts H^+^-ATPase. 

Other authors advocate the existence of single antimicrobial agents rather than a combination of distinct compounds. In this line, Miyasaki et al. [197] highlight the antibacterial role of ellagic acid from *Rosa rugosa*, a phenolic compound with a moderate effect against *Acinetobacter baumanii*. Ellagic acid is supposed to downregulate polyphosphatase kinase 1 (PPK1) gene expression as well as its activity, an enzyme with a crucial role in virulence [198]. The adjunctive use of rose hip-derived compounds is also interesting in order to potentiate the activity of existing drugs. Accordingly, tellimagrandin I extracted from *Rosa canina* enhanced the effect of the β-lactam antibiotic oxacillin against multi-drug resistant *Staphylococcus aureus* [199]. This phytochemical is able to inhibit penicillin binding protein 2′ (PBP2), a bacterial enzyme responsible to resistance to β-lactam antibiotics.

The role of *Helicobacter pylori* in peptic ulcer disease onset has been mentioned earlier [189] and the anti-ulcerogenic potential of *Rosa canina*, but the benefits from rose hip in ulcer treatment might go further. Results from Horváth et al. [200] showed that some carotenoids from *Rosa canina* had certain anti-*Helicobacter pylori* activity. They observed that one of the most abundant carotenoid was lycopene, whose antioxidant activity is a key factor in *Helicobacter pylori* eradication [201]. As ROS scavenger, lycopene prevents oxidative stress-induced DNA damage in infected gastric cells and consequently inhibits apoptosis [2]. These facts put together with the previous findings of Gürbüz et al. [187] and Lattanzio et al. [191] suggest that *Rosa canina* may be a powerful tool not only in ulcer treatment but also in its prevention, as rose hip extracts might reduce *Helicobacter pylori* burden before damage apparition.

Urinary tract infections (UTIs) are a set of disorders characterized by a high burden of one or more bacteria strain and even fungi in any part of the genitourinary tract. When not treated correctly, UTIs can promote other diseases such as pyelonephritis, especially if infectious bacteria contain urease. Urease normal function consists of urea hydrolysis to ammonium and carbon dioxide. This results in pH alkalization that promotes calcium crystals and magnesium ammonium phosphates stones formation [202,203]. Urease inhibitors are as a result an interesting drug approach to UTIs management. 

Bai et al. [204] reported the urease inhibitory effect of *Rosa indica* extracts as well as their antimicrobial effect displayed against five bacteria strain commonly found in UTIs. The authors did not delve into the concrete phytochemical that performs this inhibition, but the anti-urease activity of certain phenolic compounds has been well demonstrated [205]. Therefore, phenolic content of *Rosa indica* are the most likely urease inhibitor agents. Moreover, results from Bai et al. [204] might also be included within the previous section of “Renal disturbances”, since by preventing from crystals and stones formation they indirectly avoid nephrolithiasis or other kidney injuries. 

Gut microbiome is a complex variety of microorganisms, mainly bacteria, that colonizes the digestive tract and establishes a symbiotic relationship with the human body. Antibiotics intake or dietary changes affect microbiome composition and might lead to a situation of dysbiosis. Dysbiosis is characterized by an increase in potentially harmful bacteria accompanied by a decrease in protective microorganisms, and it is considered to be one of the main causes of intestinal disorders such as inflammatory bowel disease or colorectal cancer [206]. An ideal antibiotic would only affect pathogenic bacteria without disturbing normal colonic flora, but pharmacologic industry has not reached that stage yet. However, traditional medicine might be closer to that point than might be expected. 

*Rosa rugosa* has been proven to inhibit the growth of pathogenic bacteria but did not alter bifidobacteria or lactobacilli growth [207]. The antimicrobial effect of phenolic compounds mentioned above [208] seems to be selective, since Kamijo et al. [207] observed that tannins from *Rosa rugosa* only inhibited pathogenic bacteria growth, suggesting *Rosa rugosa* as a promising prebiotic. 

The antiviral effect of rose hip has been less investigated, but some interesting research has also been performed in this field. In 1995, the protective effect of *Rosa nutkana* extracts against bovine coronavirus was reported [65]. No further information about the anti-coronavirus effect of *Rosa nutkana* has been added up to the present, although it directed other researchers to evaluate the antiviral potential of Rose gender members.

Certain phytochemicals from *Rosa rugosa* showed anti-hepatitis C virus (HCV) activity by inhibiting its capacity to invade hepatocytes. Tellimagrandin, previously mentioned because of its enhancing effect of β-lactam antibiotics action, was able to bind two envelope proteins responsible for HCV invasive capacity [209]. The HCV invasion inhibition shown by tellimagrandin must be confirmed in vivo, but at this moment is a promising drug for hepatitis treatment. These findings bring a new point of view to our previous statements about hepatotoxicity, since the antiviral effect of rose hip would indirectly give protection from hepatic injury. 

But undoubtedly, one of the greatest viral epidemics of our time is the Acquired Immune Deficiency Syndrome or AIDS. In 2013, approximately 35 million people were infected with human immunodeficiency virus type 1 (HIV-1) and HIV-related deaths were around 1.5 million. Although pharmacological progress has greatly improved the quality-of-life of these patients, we are still quite far from AIDS eradication [210]. Current drugs inhibit distinct enzymes from the replicative life cycle of HIV in order to improve treatment efficacy. As a result, antiretroviral therapies are a combination of two or three drugs including nucleoside reverse transcriptase inhibitors, non-nucleoside reverse transcriptase inhibitors, protease inhibitors, chemokine receptor 5 (CCR5) antagonists and/or fusion inhibitors. However, therapy does not completely eradicate HIV and virus mutations are an additional complication [211]. In conclusion, there is an urgent need to develop new HIV drugs that could reduce the global burden of this disease.

*Rosa rugose* has been investigated as a source of new anti-HIV agents. A polysaccharide-peptide complex and a polymer comprised of acteoside and acteoside derivatives showed strong inhibitory effect upon viral retrotranscriptase [212]. These findings demonstrate the therapeutic potential of rose hip in HIV eradication.

The anti-mycotic effect of rose hip has also been evaluated and most relevant results are shown in Table 6, Table 7 and Table 8. More profound studies proved that this effect may be related to ursane-type triterpene glucosides content of Rose gender members [213]. 

## 4. Conclusions

Reactive oxygen species as well as nitrogen reactive species, have a key function as signalling molecules, regulating a wide variety of cellular processes. However, an imbalance between ROS and NOS levels and the antioxidant defence system leads to oxidative stress, which induces cell damage and is the main cause of different pathologies. As a result, maintaining redox balance is essential for the correct functioning of the whole organism. When ROS levels overcome the endogenous defences, mainly constituted by peroxidases and metal chelating proteins, dietary intake of exogenous antioxidants is a promising strategy for prevention and treatment6 of these ROS-dependent diseases.

In this review, *Rosa* genus has been presented as a natural source of antioxidants with a potential use in disease prevention and treatment. *Rosa* genus stands out for its high content in polyphenols (such as garlic acid, catechin and quercetin), vitamins C, E, B and carotenoids (β-carotenoid, lycopene β-cryptozanthin, rubizanthin, zeazanthin and lutein), which have an antioxidant synergy effect. The concentration of all these bioactive molecules depend on different factors such as the variety of *Rosa*, part of the plant, type of fertilization, date of harvesting, geographical origin, among others. Nevertheless, rose hip showed higher antioxidative properties than other well-known antioxidant fruits as rowanberry (*S. aucuparia*), hawthorn (*C. monogyna*), chokeberry (*A. melanocarpa*), blackcurrant (*R. nigrum*), and blueberry (*V. myrtillus*). In addition, besides its antioxidants molecules, *Rosa genus* is rich in other bioactive compounds such as unsaturated fatty acids or galactolipids, which have been proven to ameliorate different types of disease. Considering the high content of bioactive molecules contained in *Rosa* genus, rose hip has an interesting potential for the treatment of different disorders related to oxidative stress or pro-inflammatory status. 

Galactolipids contained in rose hip contributed to NO scavenging, playing an anti-tumour role. Methanol extracts and antioxidants from this plant also proved to have an antitproliferative activity in different cell lines through the disruption of ROS balance. Other mechanisms of rose hip action in cancer includes inhibition of transcription activity and reduction of cell migration.

In bone diseases, such as AR, rose hip displays a beneficial anti-inflammatory effect by reducing peripheral blood polymorphonuclear leucocytes, neutrophils and monocytes migration, demising the CRP levels, inhibiting of COX-1 and -2, reducing pro-inflammatory cytokines and chemokines and inhibiting the activation of NF-κB activation. Moreover, rose hips also play a beneficial an antioxidant role and reduce bone loss through the inhibition of osteoclastogenesis. Furthermore, in osteoporosis, antioxidants reduce loss of whole-body BMD and ascorbic acid contributes to osteoblast differentiation.

In diabetes, antioxidants and vitamin E contained in rose hip extract could ameliorate the secondary complications of this disease. *Rosa canina* has also been proven to increase proliferation of β-pancreatic cells and increase the number of pancreatic islets in vivo. Other benefits from rose hip extracts are due to its ability to inhibit α-amilases and α-glucosidades, which reduce the glucose blood levels in a similar way to α-glucosidase inhibitors currently used in diabetes type II treatment. Moreover, rose hips extracts decreased the levels of serum triglycerides and hepatic lipids whereas serum cholesterol, low-density lipoprotein, and high-density lipoprotein levels were not modified. Finally, *trans*-tiliroside contained in *Rosa canina* increased the expression of PPARα and reduced the levels of PPARγ resulting in an increase of lipid metabolism linked to a decrease in adipogenesis, and hence a reduction of abdominal visceral fat in pre-obese people. 

Due to its high content in anti-oxidative molecules, rose hip extracts have demonstrated a protective effect in other disorders associated with a disruption in redox balance such as AKI, DN, hepatotoxicity or peptic ulcer disease. Vitamins A and C, together with other flavonoids of *Rosa damascena* ameliorate convulsions in epilepsy. Moreover, flavonoid contained in *Rosa damascena* extracts, especially the flavonoid myricetin, avoid amyloid β formation and hence they can be neuroprotective in Alzheimer. Finally, antioxidants and essential oils found in *Rosa damascena* proved its potential in depression managing. Rose hip extracts have also proved to have anti-inflammatory activity, and hence they could constitute a potential therapy in diseases associated with an enhanced inflammatory response, such as AD or skin aging. 

Methanol extract *Rosa canina* had a significant antidiarrheal activity mediated by an antisecretory mechanism that reduce intestinal transit thereby increasing the absorption of water and electrolytes and inducing fluid accumulation [186].

Finally, phenols contained in rose hip extracts have an antibacterial activity, being able to increase membrane permeabilisation, nutrient deprivation and extracellular enzyme inhibition. Antiviral and antifungal properties of *Rosa* spp. have also been reported. 

Our present research has underlined the promising future of rose hip as a functional food, given its potential therapeutic role against a wide variety of diseases. The phytochemical composition of the different members of the Rose gender might promote the eradication of some of the most pressing world epidemics, as well as improve the quality-of-life of even healthy people. Moreover, single bioactive compounds of rose hip constitute a great source for new drugs which might overcome resistance-derived problems in some current drugs or even treat incurable diseases. However, it is important to notice that most of the researches that we have reviewed herein have been realized on cell culture. Therefore, the results obtained must be further confirmed in vivo. (Table 9) In this line, the most promising future for *Rosa* spp. in therapy lies in those disorders that have been tested using animal models or even human volunteers, e.g., acute kidney injury. The clinical application of *Rosa* gender members might be closer in these cases.

## Figures and Tables

**Table 1:** Anticancer activity of some *Rosa* species related to their antioxidant activity.

Rose Species	Cancer Cell Line	Reference
*Rosa canina*	Cervix epithelioid carcinoma (HeLa)	Tumbas et al. [14]
	Colon carcinoma (HT-29)	
*Rosa canina*	Non-small cell lung cancer (NCI-H460)	Guimarães et al. [85]
	Colon carcinoma (HCT-15)	
	Hepatocellular carcinoma (HepG2)	
*Rosa canina*	Colon carcinoma (Caco-2)	Jiménez et al. [21]

**Table 2:** Bioactive compounds of *Rosa* spp. and their mechanisms of action in rheumatoid arthritis.

Activity	Active Compound	Mechanism of Action	References
Anti-inflammatory	GOPO (1,2-di-*O*-*α*-linolenoyl-3-*O*-*β*-d-galactopyranosyl-*sn*-glycerol)	Reduction in peripheral blood polymorphonuclear leukocytes, neutrophils and monocytes migration Reduction in the levels of C-reactive protein (CRP)	Larsen et al. [61] Winther et al. [105] Kharazmi et al. [102]
	Fatty acids (triterpenoic acids, ursolic acid, oleanolic acid and betulinic acid)	Inhibition of COX-1 and COX-2	Jäger et al. [109] Wenzing et al. [110]
		Reduction in pro-inflammatory cytokines (TNF-α, IL-1β, IL-6, (IFN)-γ, IL-12) and chemokines CCL5 (RANTES), IP-10 (CXCL10) production	Yan et al. [106] Schwager et al. [107,108]
Inhibition of NF-κB related inflammatory response	Phytosterols β-sitosterol	Attenuation of NF-κB phosphorylation	Whang et al. [115]
	Gallic acid	Reestablishment IκBα and NF-κB association	Choi et al. [116]
	Astragalin and tormentic acid	Inhibition of IkBα phosphorylation and degradation	An et al. [117] Cheng et al. [120]
Antioxidant	Antioxidants (vitamin C, vitamin E, carotenoids, polyphenols and antioxidant enzymes)	Reduction of ROS production	Kirkeskov et al. [101] Schwager et al. [107] Jimenez et al. [21]
		Inhibition of NO release from macrophages	Kirkeskov et al. [101] Schwager et al. [107] Kim et al. [111]
		Protection against cell apoptosis, DNA and mitochondrial H_2_O_2_-induced damage and amyloid β peptide-induced oxidative injury	Choi et al. [112] Liu et al. [113]
	Aqueous extract	Inhibition of RANKL-induced osteoclastogenesis	Cheng et al. [120]

**Table 3:** Antibacterial effect of some *Rosa* species displayed as mean diameter of inhibition zone.

*Rosa* Species	Bacteria Strain	Mean Diameter of Inhibition Zone (mm)	Reference
*R. multiflora*	*E. coli*	9.0	Frey et al. [196]
	*S. typhimurium*	8.1	
*R. nutkana*	*S. aureus*	15.4	Yi et al. [193]
	MRSA	17	
	*E. faecalis*	18	
	*B. subtilis*	8	
*R. woodsii*	*S. aureus*	10	
	MRSA	9.8	
	*E. faecalis*	9	
*R. psiocarpa*	*S. aureus*	9.4	
	MRSA	7.4	
	*E. faecalis*	8.2	

**Table 4:** Antibacterial effect of some *Rosa* species displayed as minimal inhibitory concentration (MIC).

*Rosa* Species	Bacteria Strain	MIC (mg/mL)	Reference
*R. canina*	*E. coli* 8110	0.1	Kumarasamy et al. [194]
*R. rugosa*	*S. epidermis*	1.25	Olech et al. [195]
	*S. aureus*	1.25	
	*B. subtilis*	1.25	
	*M. luteus*	0.625	
	*E. coli*	1.25	
	*K. pneumoniae*	1.25	
	*P. aeruginosa*	1.25	
	*P. mirabilis*	1.25	

**Table 5:** Antibacterial effect of some *Rosa* species displayed as percentage of growth inhibition.

*Rosa* Species	Bacteria Strain	% Inhibition	Reference
*R. damascena*	*P. aeruginosa*	80.49	Talib et al. [192]
	*E. coli*	60.69	
	*S. typhimurium*	100.82	
	*B. cereus*	101.09	
	MRSA	95.75	

**Table 6:** Anti-mycotic effect of some *Rosa* species displayed as mean diameter of inhibition zone.

*Rosa* Species	Yeast Strain	Mean Diameter of Inhibition Zone (mm)	Reference
*R. nutkana*	*C. albicans*	25	Yi et al. [197]
*R. woodsii*		22.8	
*R. pisocarpa*		10	

**Table 7:** Anti-mycotic effect of some *Rosa* species displayed as minimal inhibitory concentration (MIC).

*Rosa* Species	Yeast Strain	MIC (mg/mL)	Reference
*R. rugosa*	*C. albicans*	0.156	Olech et al. [195]
	*C. parapsilosis*	0.156	

**Table 8:** Anti-mycotic effect of some *Rosa* species displayed as percentage of growth inhibition.

*Rosa* Species	Yeast Strain	% Inhibition	Reference
*R. damascena*	*C. albicans*	98.87	Talib et al. [192]
	*A. niger*	30.72	

**Table 9:** Main active compounds of *Rosa* spp. extracts and their application in human diseases.

Phytochemical	Disease of Application	Mechanism of Action	References
Ascorbic acid	Cancer	Anti-inflammatory Antioxidant	Jiménez et al. [21]
	Aging	Antioxidant and anti-inflammatory effect Contribution to skin and collagen formation	Phetcharat et al. [181]
	Arthritis rheumatoid	Inhibition of NO release from macrophages	Kirkeskov et al. [101] Kim et al. [111] Schwager et al. [108]
	Osteoporosis	Stimulation osteoblast differentiation and matrix synthesis	Devareddy et al. [124] Adom et al. [125]
Polysaccharides	Cancer	Induction of cytotoxicity in reduction of cancer metastasis by reducing the levels of matrix metalloprotease-9 (MMP-9)	Guo et al. [96] Lee et al. [97]
Polysaccharide-peptide complex and other polymers	Retroviral infection	Inhibition of viral retrotranscriptase	Fu et al. [212]
Myricetin	Alzheimer	Inhibition of amyloid β formation and tau-antagonist activity	Semwal et al. [167] DeToma et al. [168]
Quercetin	Age spots	Inhibition of tyrosinase	Fujii et al. [180]
Gallic acid	Arthritis Rheumatoid	Reestablishment IκBα and NF-κB association	Choi et al. [116]
	Diabetes	Inhibition of α-amylase and α-glucosidase	Ghadyale et al. [138]
Ellagic acid	Cancer	Anti-mutagenic and anti-carcinogenic effect	Festa et al. [29] Whitley et al. [30]
	Bacterial infections	Downregulation of polyphosphatase kinase 1 (PPK1)	Sarabhai et al. [198]
Tellimagrandin I	Bacterial infections	Inhibition of penicillin binding protein 2′ (PBP2) and enhancement of β-lactam antibiotics effect	Shiota et al. [199]
1,25-Dihydroxyvitamin D3 analogues	Acute myeloid leukemia	Induction of differentiation	Zhamanbayeva et al. [88] Corcoran et al. [89] Baurska et al. [90]
Tannin RM-3	Atopic dermatitis	Anti-inflammatory activity (reduction of the levels of cyclooxygenase 2 mediators and inducible nitric oxide synthase) Suppression Th2-polarized immune system	Wang et al. [176] Park et al. [177]
Unidentified phenols	Bacterial infections	Membrane permeabilisation, extracellular enzymes inhibition, nutrient and energy deprivation by an hyperacidification of plasma membrane interface that disrupts H^+^-ATPase	Olech et al. [195]
	Dysbiosis	Specific inhibition of pathogenic bacteria growth	Kamijo et al. [207]
	Prostate cancer	Anti-histone acetyltransferase activity	Lee et al. [91] Takayama et al. [92]
	Urinary tract infections (UTIs) and pyelonephritis	Urease inhibitor	Hassan et al. [205] Bai et al. [204]
Isoflavonoid phytoestrogen	Cancer	Stimulation of growing oestrogen dependent cells	Uifălean et al. [87]
Flavonoids	Obesity	Reduction of PPARγ expression, and prevention of lipid accumulation	Ninomiya et al. [140] Nagatomo et al. [151]
	Epilepsy	Reduction of lipid peroxidation and anticonvulsive effect	Diniz et al. [170] Homayoun et al. [171] Uttara et al. [172]
Triterpenoic acids, ursolic acid, oleanolic acid and betulinic acid	Arthritis Reumatoid	Inhibition of COX-1 and COX-2	Jäger et al. [109] Wenzing et al. [110]
Lycopene and other carotenes	Bacterial infections	Anti-*H. pylori* activity	Horváth et al. [200]
*trans*-Tiliroside	Hyperlipidaemia	Reduction of reduced plasma and liver triglyceride free fatty acid (FFA) levels. Induction of peroxisome proliferator-activated receptor α (PPAR-α) expression	Ninomiya et al. [140]
Polyunsaturated fatty acid ω-3, 6 and 9	Hyperlipidaemia	Reduction of TG synthesis, reduction of the expression of hepatic very low-density lipoprotein (VLDL)-TG, hepatic lipase and Apo CIII and increase Apo CII and VLDL-receptor	Adkins et al. [144]
Galactolipids GOPO (1,2-di-*O*-*α*-linolenoyl-3-*O*-*β*-d-galactopyranosyl-*sn*-glycerol)	Arthritis Rheumatoid	Reduction in peripheral blood polymorphonuclear leukocytes, neutrophils and monocytes migration Reduction in the levels of C-reactive protein (CRP)	Larsen et al. [61] Kharazmi et al. [102] Whinter et al. [105]
Unidentified compounds from whole extract	Arthritis rheumatoid	Inhibition of RANKL-induced osteoclastogenesis	Cheng et al. [120]
	Diabetes	Hypoglycemic effect and induction of β-cell proliferation	Taghizadeh et al. [134] Fattahi et al. [137]
	Acute kidney injury Diabetic nephropathy	Anti-inflammatory effect Reduction of ROS levels	Zhao et al. [155] Zhou et al. [157]
	Peptic ulcer	Antioxidant effect	Nazıroğlu et al. [175]
	Depression	Antioxidant effect	Nazıroğlu et al. [175]
	Antiviral	Inhibition of viral invasion capacity	McCutcheon et al. [65]
Unidentified components from fruit extract	Hepatic injuries	Reduction of peroxidation of unsaturated fatty acids	Carlo et al. [187] Meli et al. [188]
Unidentified components from root extract	Atopic dermatitis	Anti-inflammatory (reduction of cyclooxygenase 2 inducible nitric oxide synthase levels) Suppression Th2-polarized immune system	Park et al. [177]
Unidentified components from leaf extract	Diarrhoeal	Anti-secretory activity, inhibition of acetylcholine and histamine activity	Carlo et al. [187] Meli et al. [188] Rao et al. [186]
Unidentified components from rose powder	Wrinkles	Antioxidant effect: scavenging of reactive oxygen species produced by UV-radiation and decrease of matrix metalloprotease proteins levels	Phetcharat et al. [181] Jeo et al. [183]
